# Influence of the Mn_5_Ge_3_/Ge ohmic-contact interface on the Seebeck coefficient of the Mn_5_Ge_3_/Ge bilayer

**DOI:** 10.1038/s41598-023-43843-y

**Published:** 2023-10-03

**Authors:** Alain Portavoce, Siham Hassak, Maxime Bertoglio

**Affiliations:** IM2NP, Faculté des Sciences de Saint-Jérôme case 142, Aix-Marseille University/CNRS, 13397 Marseille, France

**Keywords:** Energy science and technology, Materials science, Nanoscience and technology

## Abstract

Thermoelectricity is a well-known effect that can be used to convert heat energy into electrical energy. However, the yield of this conversion is still low compared to current photovoltaic technology. It is limited by the intrinsic properties of materials, leading to intensive materials science investigations for the design of efficient thermoelectric (TE) materials. Interface engineering was shown to be a valuable solution for improving materials’ TE properties, supporting the development of multiphase TE materials. In particular, interfaces have been suggested to promote the increase of the Seebeck coefficient of materials without significantly impacting their electrical conductivity through the so-called energy filtering effect. This work aims at determining experimentally the effect of a metal/semiconductor interface exhibiting an ohmic character on the effective Seebeck coefficient of multiphase materials, focusing on the *n*-type Mn_5_Ge_3_/*p*-type Ge interface. This interface is shown not to contribute to carrier transport, but to contribute to carrier concentration filtering due to carrier injection or recombination. The Seebeck coefficient of the bi-phase material is shown to be dependent on the direction carriers are crossing the interface. The interface effect mainly results from a modification of charge carrier concentrations in the semiconductor.

## Introduction

Worldwide efforts for the development of efficient thermoelectric (TE) devices is motivated by their ability of safely produce electricity from waste heat without CO_2_ production^1–4^, theoretically ranking them among the best solutions for energy harvesting. However, their yield of converting waste heat into electrical power is still currently low (~ 10%) as it dependents on interrelated material properties exhibiting contrasting effects on TE properties^5–8^. Indeed, the conversion efficiency of a given TE material is determined by the figure of merit *ZT* = *T* × *S*^2^*σ*/*κ* (*T* the absolute temperature, *S* the Seebeck coefficient, *σ* the electrical conductivity, and *κ* the thermal conductivity)^3,5–7^. Higher the *ZT*, higher the TE efficiency, meaning that efficient TE materials should exhibit high Seebeck coefficient, high electrical conductivity, and low thermal conductivity. However, *S* and *σ* are in general inversely proportional, and the electronic component of thermal conductivity increases with electrical conductivity, limiting the TE efficiency of materials in general.

Consequently, a wide material panel is being explored for TE power generation applications, including the compounds GeTe^9,10^ and PbTe^11,12^, and half-Heusler compounds^13^, for example, aiming at discovering the most effective TE material. Though, multiphase materials were shown these past 10 years to exhibit higher *ZT* than single-phase materials^14–19^, allowing to reach *ZT* > 2 in some cases^8,18,20–22^, suggesting the possibility of reaching the limit *ZT* = 3, considered as the condition for the production of efficient devices, matching application requirements^1–3,5,23^. Usually, TE properties of multiphase materials are considered to be higher than single-phase materials due to the combination of increased phonon scattering at interfaces, increased Seebeck coefficient due to interface-mediated energy filtering, and increased electrical conductivity due to modulation doping. The effect of nanostructuration and nano-size phase inclusions on thermal conductivity has been directly proved thanks to heat conduction measurements^17,24–30^, and interpreted as due to a modification of the phonon component of thermal conductivity^5–8,18,22,23,31,32^, allowing the *ZT* numerator to be reduced. The increase of the power factor *PF* = *S*^2^*σ* (*ZT* denominator) in multiphase materials is often attributed to the effect of interfaces, in particular in the case of *S* increase, generally interpreted as the energy filtering effect of the interface between the matrix and inclusions^14,15,17–20,24,28,33,34^ or grain boundaries^27,35–39^.

Despite theoretical models^37,39–42^ and numerical calculations^18,43,44^ showing a possible increase of *S* due to interface-mediated energy filtering, the effect of interfaces on *S* still needs further experimental investigations in order to improve TE property engineering of multiphase materials. For example, the dependence of *S* with interface area in multiphase materials exhibiting enhanced *S* has been poorly studied^45^, and some experimental results shown no modification of *S* with the presence of interfaces^21,25,28,29,45–48^. Furthermore, the increase of *S* in multiphase materials has been shown as possibly due to a significant gradient of charge mobility resulting from the temperature gradient^49^. The junction between two conducting solids of very different charge carrier mobilities is thus expected to promote an increase of *S* that is not related to energy filtering of charge carriers.

The present study aims at probing the influence of a single interface on the effective Seebeck coefficient (*S*_*eff*_) of a binary multiphase material, and to provide an experimental proof of Seebeck coefficient modification due to the interface. The experiments were performed around room temperature with complementary-metal–oxide–semiconductor (CMOS) compatible materials to match the requirements of CMOS-integrated TE device for energy harvesting in portable microelectronic circuits^50,51^.

### Interfaces in multiphase thermoelectric materials

**Figure 1:**
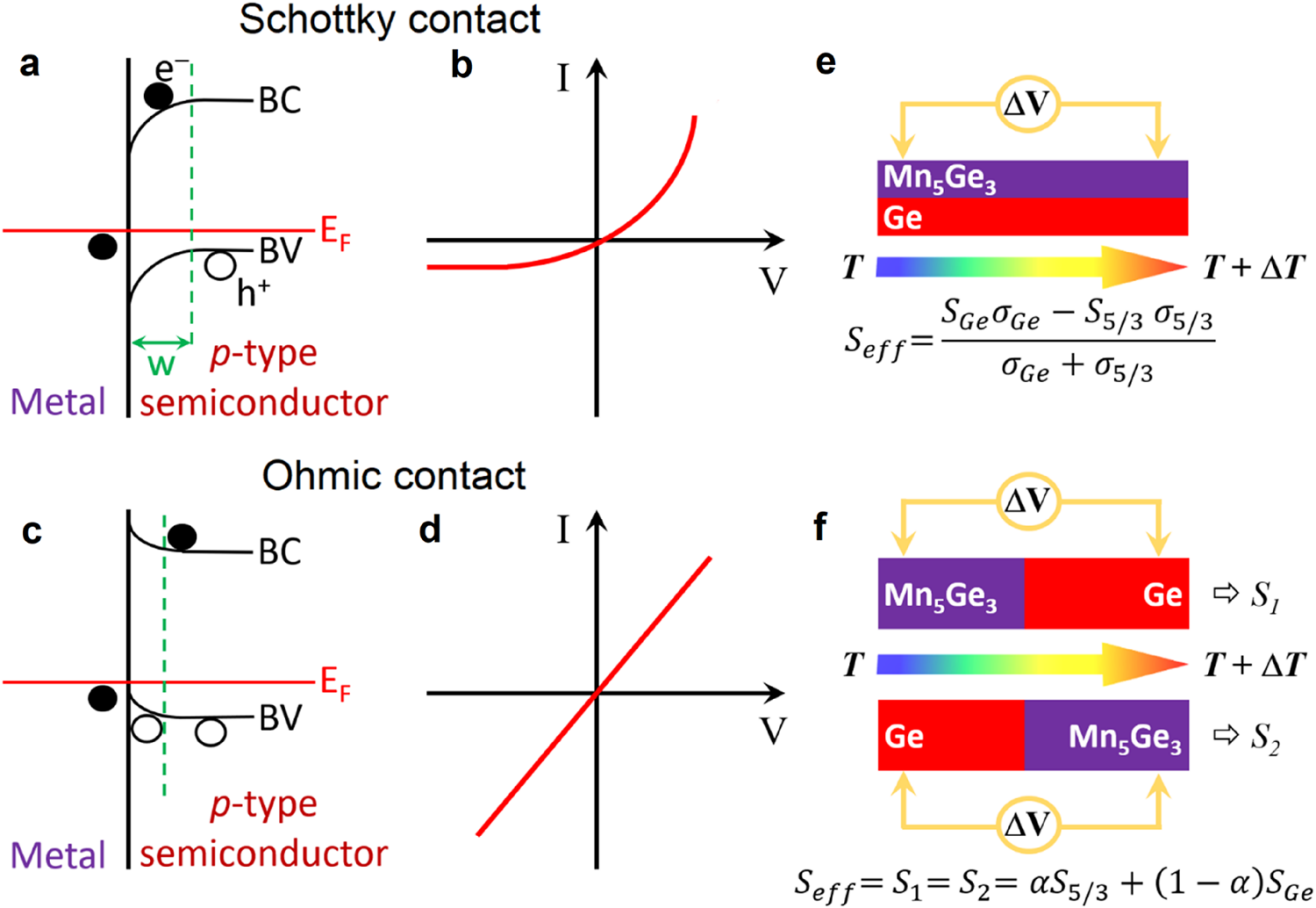
*n*-type metal/*p*-type semiconductor interfaces. Schematics of respectively the electronic band diagram and the *I*–*V* characteristics of a Schottky contact (**a**) and (**b**), and an ohmic contact (**c**) and (**d**). (**e**) and (**f**) show two types of interfaces between two materials (Mn_5_Ge_3_ and Ge) corresponding to parallel or serial charge carrier channels, respectively.

TE materials are usually *p*- or *n*-type semiconductors^28,30,46,52–54^, and Schottky contacts/interfaces between the semiconductor and a metallic phase is often considered as the main solution for interface-mediated energy filtering of charge carriers^18,37,40–42^. Figure 1a schematically presents the electronic band structure between a metallic phase and a *p*-type semiconductor, and Fig. 1b presents a schematic of a typical I–V curve of a Schottky contact. Due to the difference of Fermi level (*E*_*F*_) between the two materials before contact, an energy barrier is built (for holes in the present example) close to the interface after contact. This barrier is expected to scatter carriers of low energy, reducing the number of free carriers and increasing their average energy, leading to the increase of *S*_*eff*_ (energy filtering effect)^18,40–42^.

A second type of metal/semiconductor interface can be built, corresponding to an ohmic contact. No major barrier is present at the interface in this case (Fig. 1c), and charge carriers can cross the interface corresponding to the I-V curve schematic shown in Fig. 1d. This type of interface is usually considered as having no singular effect on *S*_*eff*_. This is nevertheless this type of interface that the present work aims at investigating, since it has been poorly studied yet and can be considered as a reference to be compared with other metal/semiconductor interfaces.

The chosen interface is the contact between a polycrystalline thin film of the metallic compound Mn_5_Ge_3_ and a polycrystalline thin film of *p*-type Ge. Mn_5_Ge_3_ is known to be *n*-type^55^ and to form an ohmic contact with Ge if Ge is *p*-type or a Schottky contact if Ge is *n*-type^56,57^. An *n*-type metal/*p*-type semiconductor interface allows possible carrier injection through the interface to be more easily evidenced. Mn and Ge were deposited at room temperature by magnetron sputtering on glass substrate^55,58^, and the Mn_5_Ge_3_/Ge bilayer was grown by reactive diffusion thanks to ex situ annealing under vacuum (*P* ~ 10^−7^ mbar) at 400 °C for 10 min^55,59,60^. The use of thin films allows the study of a single interface, as well as the volume of the phases to be precisely controlled. The sputtering technique guaranty a control of the film thickness below 5 nm, as well as a low level of contamination, below 10^19^ cm^−3^ in our case^61^. Furthermore, structural and electrical characteristics of the Mn_5_Ge_3_/Ge interface is generally considered to be well reproducible through reactive diffusion fabrication^56,62,63^.

The influence of an interface on the *S*_*eff*_ of multiphase materials can be probed through two types of geometry: (1) the parallel-phase geometry, and (2) the serial-phase geometry. In our case, the parallel-phase geometry corresponds to the Seebeck coefficient *S*_*eff*_ = Δ*V*/Δ*T* measured on the bilayer Mn_5_Ge_3_/Ge according to Fig. 1e. This geometry corresponds to the model of two conduction channels in parallel along the temperature gradient. The charge carriers are moving in the direction parallel to the interface in the two materials. Electrons in Mn_5_Ge_3_ and holes in Ge are moving towards the cold side of the bilayer. As presented in Fig. 1e, *S*_*eff*_ should only depend in this case on the Seebeck coefficients (*S*_*Ge*_ and *S*_5/3_) and the electrical conductivities (*σ*_*Ge*_ and *σ*_5/3_) of each material Ge and Mn_5_Ge_3_ if the interface has no effect. However, due to carrier confinement or due to the formation of a third phase at the interface for example, the interface can act as an additional carrier channel. In this case, the signature of the interface effect can be detected in *S*_*eff*_, as the equation given in Fig. 1e needs to be modified to take into account the Seebeck coefficient and the conductivity of this third channel located at the Mn_5_Ge_3_/Ge interface^16,44,47,64^. In the serial-phase geometry (Fig. 1f), the two materials in contact, Ge and Mn_5_Ge_3_, are placed in series in the temperature gradient: the charge carriers move perpendicularly to the interface in this case, and either electrons in Mn_5_Ge_3_ (*S*_*2*_ in Fig. 1f) or holes in Ge (*S*_*1*_ in Fig. 1f) move towards the interface if Ge or Mn_5_Ge_3_ are respectively located at the cold side. If the interface has no particular effect on carriers, *S*_*eff*_ should be equal to the sum of the two Seebeck coefficient *S*_*Ge*_ and *S*_5/3_ weighted by the relative volume fraction of their corresponding phase Ge or Mn_5_Ge_3_ (coefficient *α* in Fig. 1f), and the effective Seebeck coefficients *S*_*1*_ (measured with Ge at the hot side Fig. 1f) and *S*_*2*_ (measured with Ge at the cold side Fig. 1f) should be identical. This geometry models the possible effects of GBs or precipitates on the effective Seebeck coefficient of multiphase materials. If the Mn_5_Ge_3_/Ge interface plays a role on charge carriers, the equation given in Fig. 1f would not be fulfilled and *S*_*eff*_ should vary depending on the position of Ge (and Mn_5_Ge_3_) in the temperature gradient (in the colder region or in the hotter region), exhibiting nonreciprocity versus the temperature gradient direction^65^.

### Mn_5_Ge_3_/Ge interface contribution to carrier transport

**Figure 2:**
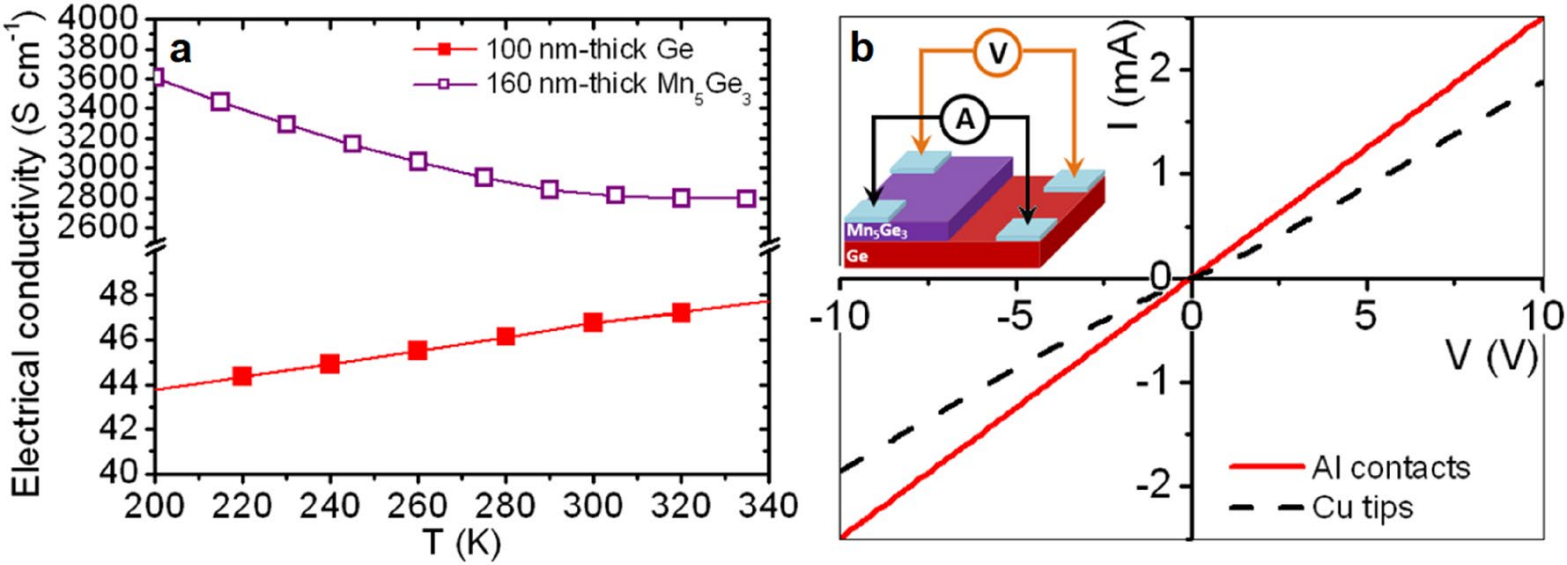
Electrical measurements performed on Ge and Mn_5_Ge_3_ thin films deposited by magnetron sputtering on glass substrate and crystallized by ex situ annealing. (**a**) Electrical conductivity of the Ge (solid squares) and Mn_5_Ge_3_ (open squares) films versus temperature. (**b**) *I*–*V* measurements performed on a Mn_5_Ge_3_-step/Ge-terrace structure (see inset) using Cu tips placed either directly on the films (dashed line) or on Al contacts sputtered on the films (solid line).

Figure 2a presents the electrical conductivity measured versus temperature (*T*) on a 100 nm-thick Ge (*σ*_*Ge*_) film and on a 160 nm-thick Mn_5_Ge_3_ film (*σ*_5/3_) deposited on glass substrate. *σ*_*Ge*_ increases with temperature, corresponding to a semiconductor behavior for Ge, while *σ*_5/3_ decreases when the temperature increases, corresponding to a metallic behavior for Mn_5_Ge_3_, as expected. The conductivity is found to be about two orders of magnitude larger in the Mn_5_Ge_3_ metallic film compared to the Ge semiconductor film. Figure 2b presents two I–V curves characteristic of the studied Mn_5_Ge_3_/Ge contact. The measurements were performed according to the Van der Pauw method on a bilayer Mn_5_Ge_3_/Ge sample, after removing part of the Mn_5_Ge_3_ film by chemical etching, making a 160 nm-thick step between a Mn_5_Ge_3_ upper terrace and a Ge lower terrace (see inset in Fig. 2b). One measurement was performed positioning four Cu tips directly on the sample (black dashed line), and one measurement was performed using the same Cu tips but positioned on Al contacts evaporated on the sample through a mask (red solid line). The I–V curves correspond to an ohmic contact, as expected.

**Figure 3:**
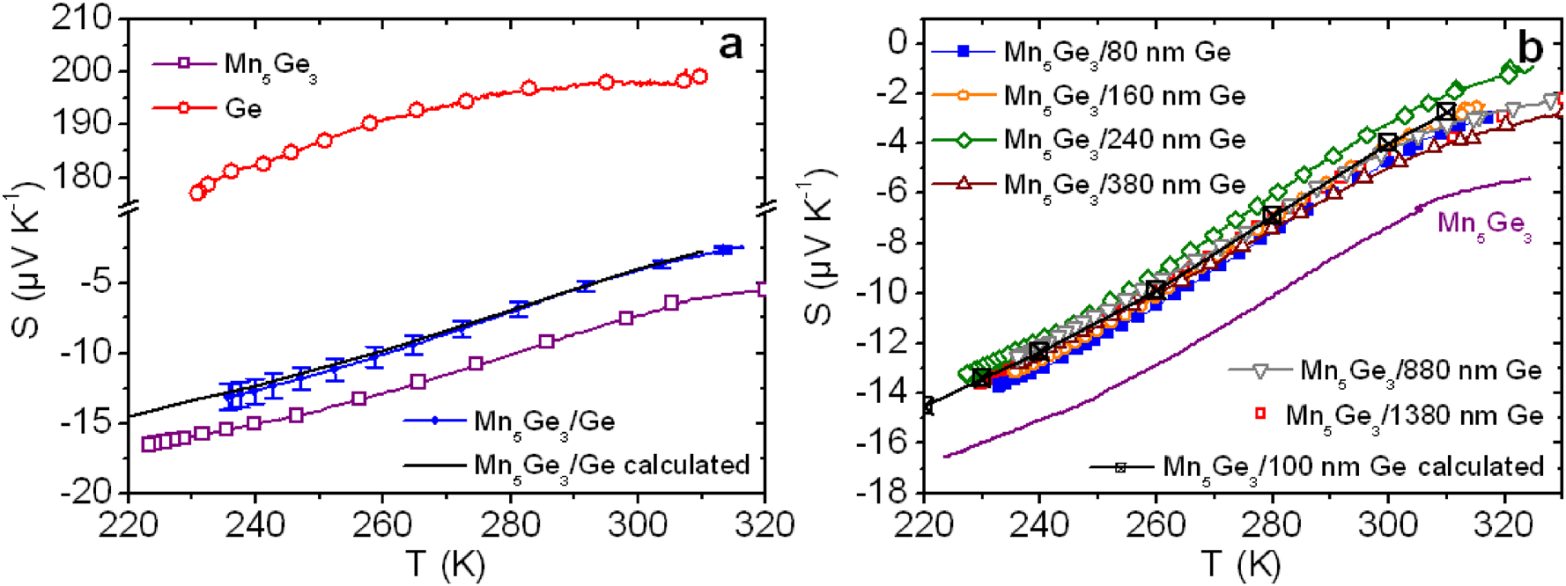
Seebeck coefficient measurements versus temperature performed on Mn_5_Ge_3_/Ge bilayers elaborated on glass substrate (see Fig. 4a). (**a**) Comparison of the Seebeck coefficient of a 160 nm-thick Mn_5_Ge_3_/100 nm-thick Ge bilayer (blue solid dots) with the Seebeck coefficient *S*_5/3_ of a 160 nm-thick Mn_5_Ge_3_ film (purple open squares), *S*_Ge_ of a 100 nm-thick Ge film (red open circles), and the theoretical effective coefficient *S*_*eff-th*_ of the bilayer (black solid line) calculated using the equation in Fig. 1e and the experimental values of *S*_5/3_ and *S*_Ge_, as well as of *σ*_5/3_ and *σ*_Ge_ presented in Fig. 2a. (**b**) Comparison between the Seebeck coefficients of different Mn_5_Ge_3_/Ge bilayers made of a same 160 nm-thick Mn_5_Ge_3_ film in contact with a Ge film of different thickness, from 80 to 1380 nm. The purple solid line and the black crossed squares respectively correspond to *S*_5/3_ and *S*_*eff-th*_, also shown in (**a**).

Figure 3a shows the Seebeck coefficients *S*_*Ge*_ (red open circles) of the 100 nm-thick Ge film and *S*_5/3_ (purple open squares) of the 160 nm-thick Mn_5_Ge_3_ film versus temperature. *S*_*Ge*_ > 0 and increases with temperature, while *S*_5/3_ < 0 and decreases when temperature increases. The opposite sign of *S*_*Ge*_ and *S*_5/3_ confirms that the Mn_5_Ge_3_/Ge ohmic contact (Fig. 2b) is obtained between *n*-type metallic Mn_5_Ge_3_ and *p*-type semiconductor Ge (Fig. 2a). Electrons and holes are respectively the majority carriers in the Mn_5_Ge_3_ film and the Ge film, moving towards the colder region of the films. According to Hall effect measurements, the average carrier concentration in the Mn_5_Ge_3_ film is ~ 2.0 × 10^20^ cm^−3^, while it is ~ 9.0 × 10^18^ cm^−3^ in the Ge film at room temperature. The carrier concentration is thus one order of magnitude smaller in the Ge film, while ∣*S*_*Ge*_ ∣ is more than one order of magnitude larger than ∣*S*_5/3_∣.

**Figure 4:**
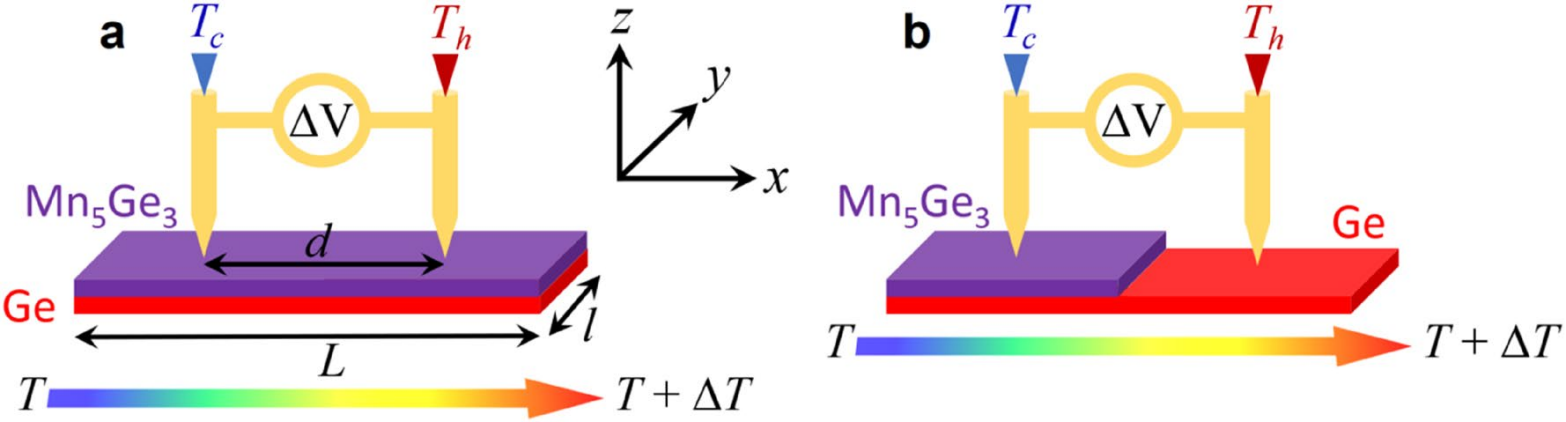
Schematics describing the two geometries used for Seebeck coefficient measurements aiming at investigating the Mn_5_Ge_3_/Ge interface contribution to the Seebeck coefficient in the case of parallel (**a**) or serial (**b**) charge carrier channels. In (**b**), the temperature gradient is applied in the direction *x* perpendicular to the Mn_5_Ge_3_-step.

*S*_*Ge*_ and *S*_5/3_ are compared in Fig. 3a with *S*_*eff*_ (blue dots) of the 160 nm-thick Mn_5_Ge_3_/100 nm-thick Ge bilayer measured according to the parallel-phase geometry. Figure 4a shows a schematic of experimental conditions. The value of *S*_*eff*_ is in between of those of *S*_*Ge*_ and *S*_5/3_, but it is negative in the investigated temperature range and is close to *S*_5/3_. *S*_*eff-th*_ calculated using the equation given in Fig. 1e and the experimental values of *σ*_*Ge*_ and *σ*_5/3_ (Fig. 2a) and of *S*_*Ge*_ and *S*_5/3_ (Fig. 3a) versus temperature is also shown in Fig. 3a (black solid line). *S*_*eff-th*_ is in very good agreement with experimental *S*_*eff*_, showing no contribution of the Mn_5_Ge_3_/Ge interface. *S*_*eff*_ is closer to *S*_5/3_ than to *S*_*Ge*_ due to *σ*_5/3_ >> *σ*_*Ge*_ (Fig. 2a). Figure 3b presents the influence of the Ge layer thickness *t*_*h*_ on experimental *S*_*eff*_ compared to *S*_*eff-th*_ calculated using experimental *σ*_*Ge*_ and *S*_*Ge*_ measured on a 100 nm-thick Ge film. The thickness of the Mn_5_Ge_3_ layer was kept constant (160 nm), while the thickness of the Ge layer was varied between 80 and 1380 nm in six different Mn_5_Ge_3_/Ge bilayers. All the measurements are close to *S*_*eff-th*_, the maximum difference between *S*_*eff*_ and *S*_*eff-th*_ at *T* = 300 K being 22% for example. Furthermore, no obvious dependence of the Ge thickness on *S*_*eff*_ could be determined, *S*_*eff*_ being closer to *S*_*eff-th*_ in the sample with *t*_*h*_ = 880 nm than in the sample with *t*_*h*_ = 240 nm for example.

These results show that the interface between two polycrystalline thin films of Mn_5_Ge_3_ and Ge corresponding to an ohmic contact does not provide a conduction channel to charge carriers. The experimental *S*_*eff*_ of the Mn_5_Ge_3_/Ge bilayer can be predicted independently of layer thicknesses using the model of parallel conduction channels along the temperature gradient (Fig. 1e) for Ge and Mn_5_Ge_3_ layers thicker than 80 nm.

### Mn_5_Ge_3_/Ge interface contribution to carrier filtering

**Figure 5:**
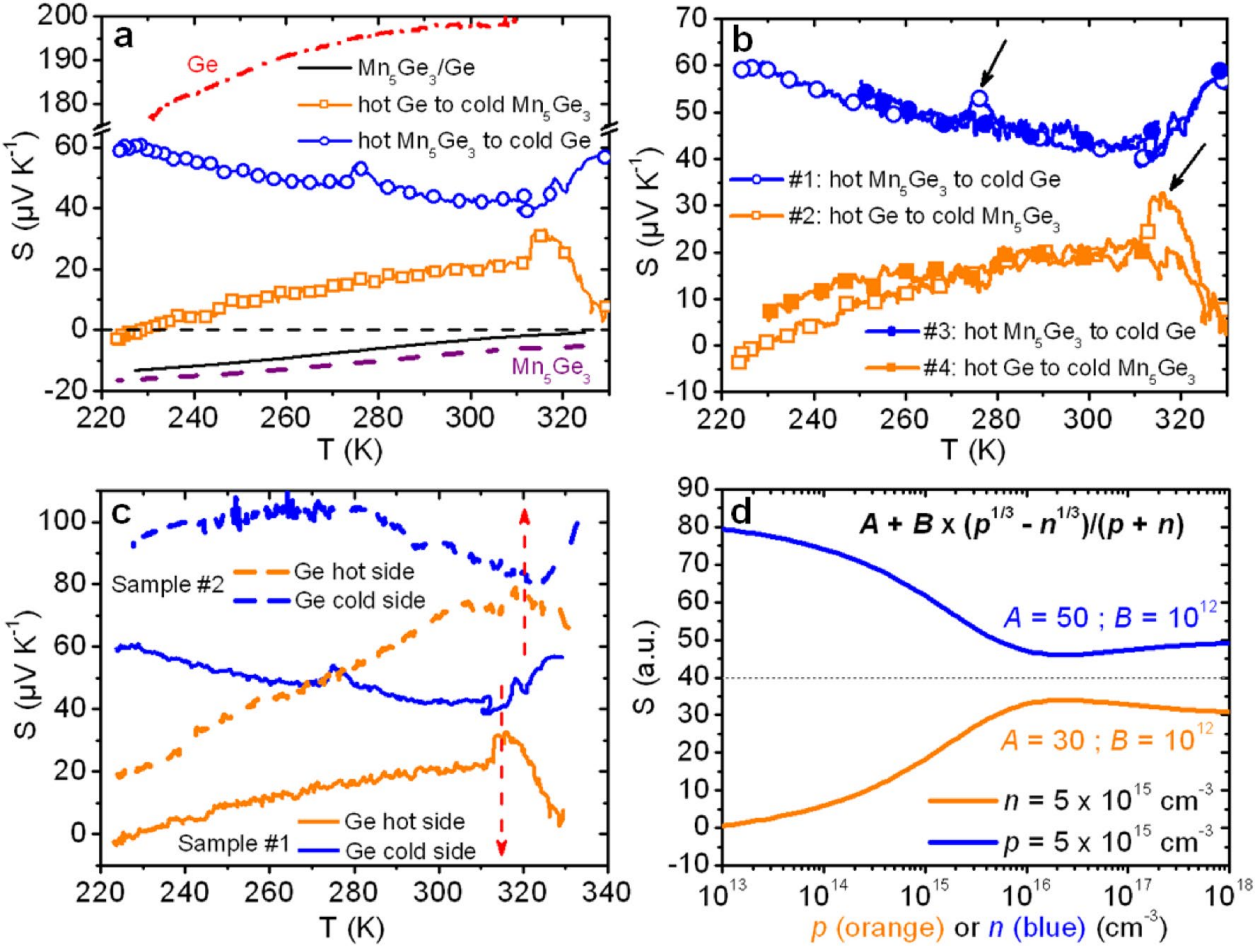
Seebeck coefficient variations versus temperature of Mn_5_Ge_3_-step/Ge-terrace structures (see Fig. 4b). (**a**) Seebeck coefficient measured for both cases: the Ge side placed in the hot (orange open squares) or in the cold (blue open circles) region of the temperature gradient, compared to the Seebeck coefficient of a single Mn_5_Ge_3_ (purple dashed line) or Ge (red dashed dotted line) film, and a Mn_5_Ge_3_/Ge bilayer (black solid line). (**b**) Four different measurements performed on a same Mn_5_Ge_3_-step/Ge-terrace sample, measurements #1 and #3 with Ge in the cold region of the temperature gradient, measurements #2 and #4 with Ge in the hot region of the temperature gradient. (**c**) Same measurements as in (**b**) but performed on two different Mn_5_Ge_3_-step/Ge-terrace samples. (**d**) Variations of the fraction (*p*^1/3^ − *n*^1/3^)/(*p* + *n*) versus *p* (orange solid line) or *n* (blue solid line).

In order to probe the contribution of the Mn_5_Ge_3_/Ge interface in the serial-phase geometry, *S*_*eff*_ was measured on Mn_5_Ge_3_/Ge bilayer samples exhibiting a step between the Mn_5_Ge_3_ layer and the Ge film as presented in Fig. 4b (Mn_5_Ge_3_-step/Ge-terrace structure). Δ*V* was measured across the Mn_5_Ge_3_ step while the Ge side of the sample was located either in the colder part or the hotter part (as in Fig. 4b) of the temperature gradient. Figure 5a presents the Seebeck coefficient measured versus temperature through the Mn_5_Ge_3_/Ge interface with the Ge side located either in the cold (blue open circles) or the hot region (orange open squares) of the temperature gradient. The Seebeck coefficient is different from the single films of Ge and Mn_5_Ge_3_, as well as from that measured on the Mn_5_Ge_3_/Ge bilayer (Fig. 5a), and cannot be reproduced using the regular equation of the serial-phase geometry given in Fig. 1f. Furthermore, at the same temperature, the Seebeck coefficient is different when charge carriers cross the interface from Ge-to-Mn_5_Ge_3_ (hot Ge) or from Mn_5_Ge_3_-to-Ge (cold Ge), showing clearly an impact of the interface on *S*_*eff*_. *S*_*eff*_ exhibits higher values if the carriers move from hot Mn_5_Ge_3_ to cold Ge, and depending on the carrier direction through the interface, *S*_*eff*_ follows opposite variations versus temperature (increasing or decreasing versus temperature), exhibiting almost a symmetrical behavior around the value of ~ 30 µV K^−1^. Figure 5b shows four consecutive Seebeck measurements performed on the same Mn_5_Ge_3_-step/Ge-terrace sample. The measurements are reproducible. However, few differences in the Seebeck values are observed between same-condition measurements (arrows in Fig. 5b), the interface influence on carriers displaying a stochastic character.

Figure 5c presents Seebeck measurements performed on two different Mn_5_Ge_3_-step/Ge-terrace samples. The global behavior of *S*_*eff*_ versus temperature is the same in the two samples depending on the carrier diffusion direction toward the interface. Though the values of the Seebeck coefficient are different for the two samples at the same temperature. *S*_*eff*_ is about 20 to 40 µV K^−1^ higher in the sample #2 (Fig. 5c). As shown in Fig. 1f, the effective Seebeck coefficient should obey *S*_*eff*_ = *α S*_5/3_ + (1 − *α*) *S*_Ge_ in the case of the Mn_5_Ge_3_/Ge serial-phase geometry. However, the bipolarity of the Ge film should be considered. Indeed, the Ge film being extrinsic, it contains two different charge carriers of significantly different concentrations: holes (*h*) and electrons (*e*), being respectively the majority and the minority charge carriers in the present case. Thus, a similar model as for the parallel-phase geometry should be used to consider the two conduction channels of holes and electrons in Ge, giving:

1 $$ S_{eff} = \alpha S_{5/3} + \left( {1 - \alpha } \right)\frac{{S_{h} \sigma_{h} - S_{e} \sigma_{e} }}{{\sigma_{h} + \sigma_{e} }} $$

With *S*_*h*_ and *S*_*e*_ the respective Seebeck coefficients of holes and electrons, and *σ*_*h*_ and *σ*_*e*_ their respective conductivities in the Ge film (*σ*_*Ge*_ = *σ*_*h*_ + *σ*_*e*_). Typically, the coefficients *α* and (1−*α*) respectively correspond to the volume fractions of the two phases Mn_5_Ge_3_ and Ge in the considered bi-phase material. Though these coefficients should describe the volume difference of Mn_5_Ge_3_ and Ge crossed by the charge carriers between the two electrodes placed on the two sides of the Mn_5_Ge_3_ step considering the measurement geometry shown in Fig. 4b. According to Eq. (1), *S*_*eff*_ depends on (1) the position of the electrodes during measurement (i.e. the distance between the Mn_5_Ge_3_ step and the electrode located on the Ge terrace for example, Fig. 4b) through the coefficient *α*, (2) the variations of *S*_5/3_ versus temperature, and (3) the Ge bipolarity versus temperature, without considering any interface effect.

The interface is an extended defect that can hold interfacial carrier traps, as well as carrier recombination centers^66–69^. Furthermore, the *n*-type Mn_5_Ge_3_/*p*-type Ge interface corresponds to an ohmic contact (Fig. 2b), and should provide carrier injection between Mn_5_Ge_3_ and Ge. Consequently, the interface can act as a sink (carrier recombination) or a source (carrier injection) of charge carriers in the Mn_5_Ge_3_ and Ge films depending on the carrier flux direction towards the Mn_5_Ge_3_/Ge interface. The Mn_5_Ge_3_/Ge interface is expected to locally modify the concentration of the charge carriers, and thus, to have a combined effect on the five parameters *S*_5/3_, *S*_*h*_, *S*_*e*_, *σ*_*h*_, and *σ*_*e*_ in Eq. (1), leading to complex variations of *S*_*eff*_ versus temperature, since temperature plays differently on carrier trapping/releasing, carrier recombination, and carrier injection. Neglecting the effect of the interface on the concentration of electrons in metallic Mn_5_Ge_3_, and assuming that the main effect of the interface is the modification of the hole concentration (*p*) and the electron concentration (*n*) in the semiconductor, one can roughly assume *S*_*h*_ ∝ *p*^−2/3^, *S*_*e*_ ∝ *n*^−2/3^^5–8,30,32,44^, *σ*_*h*_ ∝ *p*, and *σ*_*e*_ ∝ *n*^6,30,55^, leading to the approximation

2 $$ S_{eff} \propto S_{Ge} \propto \frac{{p^{1/3} - n^{1/3} }}{p + n} $$

Figure 5d presents the variation of Eq. (2) either if *p* increases from 10^13^ to 10^18^ cm^−3^ while *n* = 5 × 10^15^ cm^−3^ (orange solid line) or if *n* increases from 10^13^ to 10^18^ cm^−3^ while *p* = 5 × 10^15^ cm^−3^ (blue solid line). The plot uses two arbitrary constant *A* and *B* according to *S* = *A* + *B* (*p*^1/3^ − *n*^1/3^)/(*p* + *n*), which were adjusted in order to obtain Seebeck coefficient variations of the same order as those in Fig. 5c. The Seebeck coefficient variations with temperature suggested by Eq. (2) are not the same as the experimental variations reported in Fig. 5c, but show similar trends despite strong simplifications, particularly concerning *p* and *n* variations (Fig. 5d). The opposite variations of *S*_*eff*_ with opposite carrier diffusion directions toward the interface reported in Fig. 5 can be interpreted as mostly resulting from the modification of the charge carrier concentrations in Ge versus temperature due to the presence of the interface. *S*_*eff*_ variations when the charge carriers cross the interface from Mn_5_Ge_3_-to-Ge (cold Ge) can be interpreted as an increase of the fraction *n*/*p* versus *T*, while *S*_*eff*_ variations when the charge carriers cross the interface from Ge-to-Mn_5_Ge_3_ (hot Ge) can be interpreted as a decrease of the fraction *n*/*p* versus *T*.

**Figure 6:**
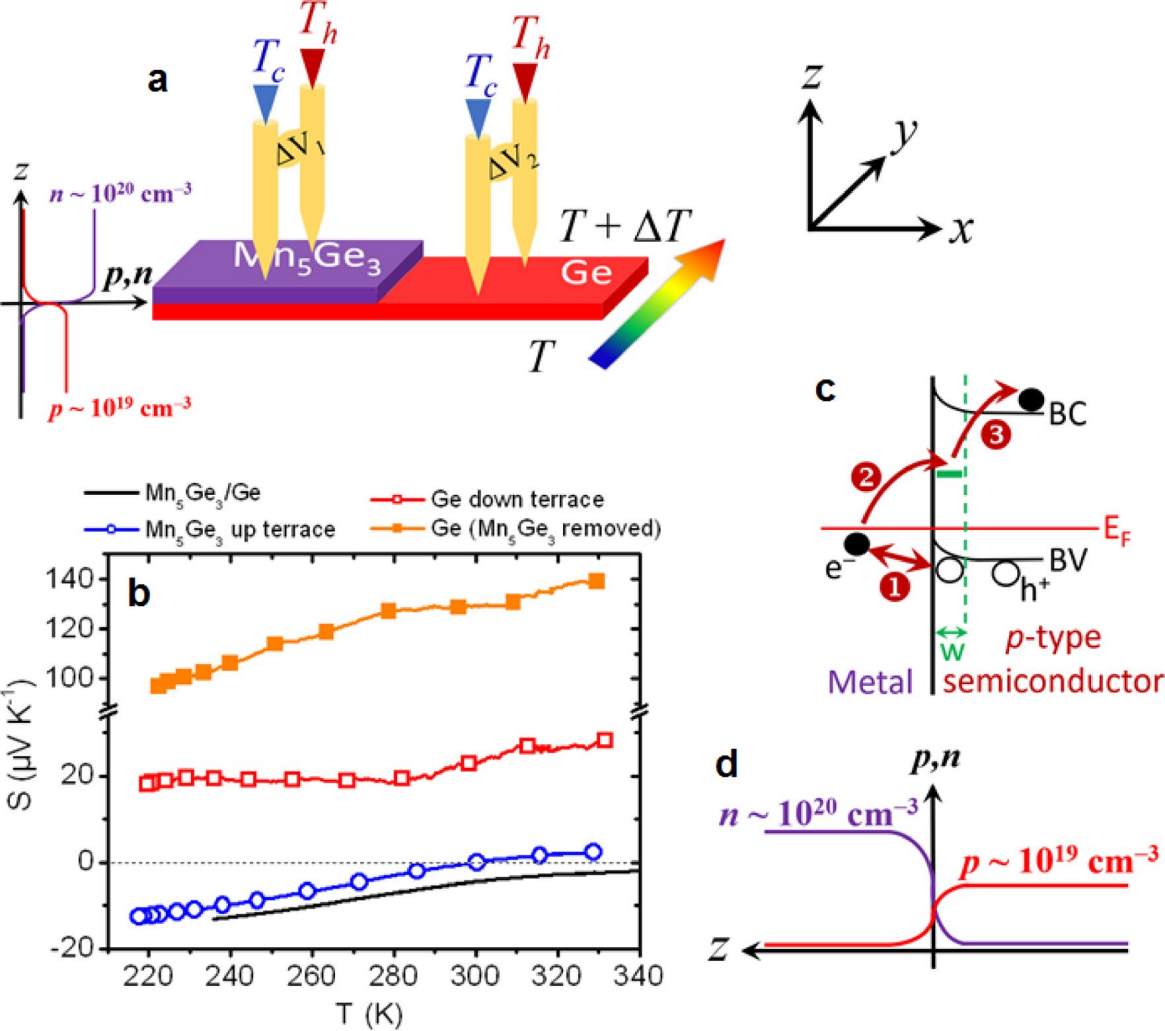
Seebeck coefficient measurements versus temperature performed on a same Mn_5_Ge_3_-step/Ge-terrace sample. (**a**) Schematic of the measurement geometry, the temperature gradient is applied in the direction *y* parallel to the Mn_5_Ge_3_-step. (**b**) Comparison between the Seebeck coefficients measured on the Mn_5_Ge_3_/Ge bilayer before the formation of the Mn_5_Ge_3_-step by chemical etching (black solid line, see Fig. 4a), and on both the Mn_5_Ge_3_ upper terrace (blue open circles, Δ*V*_1_/Δ*T* in **a**) and the Ge down terrace (red open squares, Δ*V*_2_/Δ*T* in (**a**) after the formation of the Mn_5_Ge_3_-step, and finally on the same Ge film once the Mn_5_Ge_3_ layer has been chemically removed (orange solid squares). (**c**) Schematic of the electronic band diagram at the Mn_5_Ge_3_/Ge interface during the Seebeck measurements sketched in (**a**). (**d**) Schematic illustrating the expected variations of the free electron concentration *n* and free hole concentration *p* through the Mn_5_Ge_3_/Ge interface, along the *z* direction (see also **a**).

Figure 6a presents a third type of measurements that was performed in order to support this interpretation. Two effective Seebeck coefficients were measured on a same Mn_5_Ge_3_-step/Ge-terrace sample while the temperature gradient was oriented along the Mn_5_Ge_3_ step. A first measurement was performed on the Mn_5_Ge_3_ step, and a second was performed on the Ge terrace. The first measurement should be similar to *S*_*eff*_ measured on Mn_5_Ge_3_/Ge bilayers (Fig. 3), and the second should correspond to *S*_*Ge*_ (red open circles in Fig. 3a) assuming no interface effect. Figure 6b shows four Seebeck coefficient measurements acquired sequentially versus temperature on the same Mn_5_Ge_3_/Ge bilayer. A first measurement was performed as described in Fig. 4a (black solid line), before the fabrication of a Mn_5_Ge_3_ step by chemical etching. *S*_*eff*_ < 0 in the investigated temperature range and its variations with temperature are similar to the previous measurements performed on similar samples, reported in Fig. 3. Then, a Mn_5_Ge_3_ step was made by chemical etching on the same sample, and *S*_*eff*_ was measured on the Mn_5_Ge_3_ step (blue open circles, *S*_*eff*_ = Δ*V*_*1*_/Δ*T* in Fig. 6a) and on the Ge terrace (red open squares, *S*_*eff*_ = Δ*V*_*2*_/Δ*T* in Fig. 6a) of the Mn_5_Ge_3_-step/Ge-terrace sample.

One can note that *S*_*eff*_ measured on the Mn_5_Ge_3_ step on the Mn_5_Ge_3_-step/Ge-terrace sample is slightly smaller than the measurements performed on the Mn_5_Ge_3_/Ge bilayer. In particular, *S*_*eff*_ change sign close to room temperature on the Mn_5_Ge_3_ step. According to the equation in Fig. 1e, the modification of *S*_*eff*_ can result from the modification of the four parameters *S*_5/3_, *S*_*Ge*_, *σ*_5/3_, and *σ*_*Ge*_. Consequently, the interpretation of this result is not straightforward, as it can result from carrier concentration variations in both Mn_5_Ge_3_ and Ge. Though the difference Δ*S*_*eff*_ between the Seebeck coefficients measured on the Mn_5_Ge_3_/Ge bilayer and on the Mn_5_Ge_3_ step on the Mn_5_Ge_3_-step/Ge-terrace sample at the same temperature is rather small: Δ*S*_*eff*_ < 5 µV K^−1^. In contrast, the Ge Seebeck coefficient measured on the Ge terrace of the Mn_5_Ge_3_-step/Ge-terrace sample is significantly smaller than that measured on the 100 nm-thick Ge film (red open circles in Fig. 3a). The interface has a strong effect on bipolar Ge as formerly suggested (Eq. 2). *S*_*Ge*_ < 30 µV K^−1^ in the Ge terrace of the Mn_5_Ge_3_-step/Ge-terrace sample in the investigated temperature range, corresponding to a decrease of *S*_*Ge*_ of about 160 µV K^−1^ with the presence of the interface. Finally, the Mn_5_Ge_3_ step was entirely removed by chemical etching, leaving only a Ge layer on the glass substrate and the Ge Seebeck coefficient was again measured on this sample (orange solid squares in Fig. 6b). A rather higher Ge Seebeck coefficient is recovered without the interface, with *S*_*Ge*_ ≥ 100 µV K^−1^. The fact that *S*_*Ge*_ is not as high as initially in the case of the unetched 100 nm-thick Ge film is attributed to an effect linked to the sequential chemical etching and sample annealing during the Seebeck measurements.

Figure 6c shows a schematic of the expected electronic band diagram at the Mn_5_Ge_3_/Ge interface. The temperature gradient being oriented in the *y* direction during measurement (Fig. 6a), the electro-chemical potential at the interface between Mn_5_Ge_3_ and Ge should be at equilibrium at each position *y* along the temperature gradient. Nevertheless, because of the opposite types of the Mn_5_Ge_3_ (*n*-type) and Ge (*p*-type) films, a strong electron and hole concentration gradient should be present at the interface vicinity (Fig. 6d), which can act as the driving force for carrier injection and electron–hole recombination. For example, interfacial electron–hole recombination (mechanism (1) in Fig. 6c) should act as a sink of electrons in Mn_5_Ge_3_ and a sink of holes in Ge, while interfacial carrier injection should promote the injection of holes in Mn_5_Ge_3_ and the injection of electrons in the Ge valence band due to the ohmic property of the contact (mechanism (1) in Fig. 6c). Electron injection in the Ge conduction band due to the presence of defects at the interface is also possible depending on temperature (mechanisms (2) + (3) in Fig. 6c). However, the electron concentration in Mn_5_Ge_3_ being about one order of magnitude higher than the hole concentration in Ge according to Hall effect measurements (Fig. 6d), interfacial carrier injection and recombination are expected to have a stronger effect on the carrier concentrations in Ge, in agreement with the results displayed in Figs. 5 and 6b.

### Outlook

The influence of the *n*-type Mn_5_Ge_3_/*p*-type Ge interface on the effective Seebeck coefficient of a Mn_5_Ge_3_/Ge bilayer has been investigated experimentally. The Mn_5_Ge_3_/Ge interface does not contribute to charge carrier transport. However, this metal/semiconductor interface of ohmic character contributes to charge carrier filtering. The filtering effect results from carrier injection or recombination at the interface, which depends on the direction of the carrier flux. The charge carrier concentration in the metal being significantly higher than the charge carrier concentrations in the semiconductor, interfacial carrier injection and recombination have a stronger effect in the semiconductor. Consequently, the modification of the effective Seebeck coefficient of the multiphase material due to the presence of the interface is mainly driven by the change of carrier concentrations in the semiconductor. The Seebeck coefficient of the Mn_5_Ge_3_/Ge bi-phase material is driven by either an increase or a decrease of the fraction *n*/*p* in Ge, depending if the carrier flux respectively crosses the interface from Mn_5_Ge_3_-to-Ge or from Ge-to-Mn_5_Ge_3_. Ohmic metal/semiconductor interfaces alter the Seebeck coefficient of semiconductor–metal multiphase materials through carrier concentration filtering, mainly effective in the semiconductor volume.

## Materials and methods

The Ge and Mn_5_Ge_3_ films were elaborated by magnetron sputtering and solid-state reactive diffusion. 99.99% pure Ge and 99.9% pure Mn targets were sputtered on *l* = 1.5 × *L* = 2.5 cm^2^ (Fig. 4a) glass substrates in a commercial magnetron sputtering system with a base vacuum of 10^−8^ Torr^55,58,59^. Ge and Mn deposition rates were calibrated by measuring the thickness of different films deposited in different conditions on oxidized Si substrate using X-ray reflectivity. The substrates were cleaned 10 min in an acetone bath before to be rinsed 10 min in alcohol in an ultrasonic cleaner. They were finally kept 30 min at 423 K in a baking furnace, before to be loaded in the sputtering chamber. Ge and Mn were deposited sequentially at room temperature on the glass substrates to form a Mn/Ge bilayer on the glass substrate. The samples were ex situ annealed at 400 °C for 10 min under vacuum (*P* ~ 10^−7^ mbar) after deposition allowing for the growth of the Mn_5_Ge_3_ layer via reactive diffusion and the full crystallization of the Ge layer^55,59,70^. The thickness of the Mn layer was maintained the same for all the samples, while the thickness of the Ge film was varied in order to obtain a 160 nm-thick Mn_5_Ge_3_ layer on a Ge layer of different thicknesses. Hall measurements and sample resistivity were measured in the Van der Pauw geometry using a lab-made setup operating between 20 and 350 K. The applied magnetic field for Hall measurements was 0.5 T. The Seebeck coefficients of the films were measured using a home-made setup^48,53,55,71^ between *T* = 225 and 325 K. The distance *d* between the two electrodes allowing to simultaneously determine the potential difference Δ*V* as well as the temperature gradient Δ*T* = *T*_*h*_ − *T*_*c*_ was 1 cm (Fig. 4a).

## Data Availability

All data are available in the main text.
